# Acute Upper Gastrointestinal Bleeding in Hexagenerians or Older (≥60 Years) Versus Younger (<60 Years) Patients: Clinico-Endoscopic Profile and Outcome

**DOI:** 10.7759/cureus.13521

**Published:** 2021-02-23

**Authors:** Rajpal S Yadav, Payal Bargujar, Hans R Pahadiya, Rahul K Yadav, Jitendra Upadhyay, Alok Gupta, Manoj Lakhotia

**Affiliations:** 1 Internal Medicine, Dr. Sampurnanand Medical College, Jodhpur, IND; 2 Pediatrics, Sawai Man Singh Medical College, Jaipur, IND; 3 Medicine, Sawai Man Singh Hospital, Jaipur, IND; 4 Medicine, Sawai Man Singh Medical College, Jaipur, IND

**Keywords:** upper gastrointestinal bleeding, varices, co-morbidity, mortality, re-bleed

## Abstract

Background and aims

Acute upper gastrointestinal (UGI) bleeding is one of the serious and potentially life-threatening medical emergencies, causing significant mortality and morbidity. This study aimed to evaluate the clinico-endoscopic profile and outcome among patients aged <60 years who presented for UGI bleeding compared to those aged ≥60 years.

Methods

This prospective observational study was conducted among 194 patients who presented with symptoms or signs of UGI bleed. All patients were divided into two groups, group A (age <60 years), and group B (age ≥60 years). UGI endoscopy was performed using Olympus N19 Endoscope. Rockall scoring (RS) system and Glasgow Blatchford score (GBS) were used to predict the prognosis and re-bleeding.

Results

Of the total, group A included 150 (77.31%) patients and group B 44 (22.69%) patients. The most common presentation was hematemesis and melena in both groups, whilst isolated hematochezia was more common in group A (6.67%, vs. 2.27%, p>0.05). The main cause of bleeding was a variceal bleed in both groups, but it was significantly higher in group A patients (p<0.05). Elderly patients had a significantly higher number of peptic ulcer and malignancy-related bleed (p<0.05). Group A patients had a significantly higher proportion of patients with tachycardia (45.33%, vs. 27.27%, p<0.05), shock (43.33% vs. 13.63%, p<0.05), pallor (76.66% vs. 56.81%, p<0.05), and blood transfusion requirement (64% vs. 45.45%, p<0.05) as compared to group B. Thirty days re-bleeding and mortality rate were similar in both the groups. RS in both groups was 5.02±2.12 vs. 5.98±1.91, p>0.05. GBS was 11.65±4.61 vs. 10.68±4.65, p>0.05. Mortality was significantly higher in patients with RS ≥6 and GBS ≥10.

Conclusion

This study concluded variceal bleeding as a predominant cause of UGI bleed in both age groups, and it was significantly higher in younger. Interestingly, younger patients were more hemodynamically unstable, probably due to the presence of more severe anemia, shock, and hematochezia. The presence of multiple co-morbidities in both the group kept the 30 days mortality and re-bleed rates similar.

## Introduction

Acute upper gastrointestinal (UGI) bleeding is one of the common life-threatening medical emergencies worldwide, having a mortality of around 2-10% [1]. UGI bleeding includes bleeding from the esophagus, stomach, and duodenum up to the ligament of treitz. UGI bleed is common in the male population and the annual incidence ranges from 50 to 150/100,000 population with increasing prevalence in aged people [2-4]. The mortality and morbidity because of UGI bleed is a major concern and mainly depends on patients’ demographic profile, cause of bleed, and timely management of the same. Patients above the age of 60 years represent 35-45% of all patients presenting with UGI bleed [5-8]. Elderly patients have more chances of deterioration after UGI bleed due to multiple comorbidities, underlying preexisting diseases, or a history of chronic nonsteroidal anti-inflammatory drug (NSAID) or antiplatelet use. The usual presentation of UGI bleed is hematemesis and/or melena or hematochezia [9]. Endoscopic and medical management, including terlipressin, octreotide, and proton-pump inhibitors are the mainstay of treatment. Despite many recent advances in diagnostic and treatment modalities of UGI bleed, re-bleed and mortality rates are still high, the in-hospital mortality rate is high (13%), and re-bleeding is common (15%) [3,10,11].

In India, the portal hypertension-related variceal bleed was found to be the most common cause of UGI bleed [12,13]. In contrast in another study from the Orisa state of India, the duodenal ulcer was found to be the most common [14]. We herein aimed to study the clinico-endoscopic profile and UGI bleeding outcomes in patients of age <60 years as compared to the older ones (≥60 years).

## Materials and methods

This was a prospective observational study conducted in the Department of Medicine and Gastroenterology of Dr. S.N. Medical College, Jodhpur, India, from January 2015 to 2016. The study was approved by the ethical committee of the College.

The inclusion criterion was patients aged >12 years with the presentation of symptoms or signs of UGI bleed who gave informed or written consent.

The exclusion criteria were patients who had bleeding due to road traffic accident, had contraindications for endoscopy, or who were unwilling to participate in the study.

All these patients were divided into two groups according to their age; group A - patients with age <60 years, and group B with age ≥60 years. Their detailed clinical history, demographic profile, and examination findings, including vital parameters were noted. All necessary lab investigations required for diagnosing the cause of UGI bleed and for co-morbidities were done. These included hemogram, renal function test, liver function tests, prothrombin time and international normalized ratio, blood grouping, ultra-sonography, chest X-ray, ECG, etc. Blood transfusion and other blood products were given when required. After the patient became hemodynamically stable, a UGI endoscopy was performed using Olympus N 19 Endoscope. Rockall scoring (RS) system and Glasgow Blatchford score (GBS) were used to predict the prognosis and re-bleeding [15,16]. Parameters, i.e., age, initial heart rate, systolic blood pressure, melena or syncope, hemoglobin, blood urea nitrogen, coexistent hepatic disease, heart failure, or other significant comorbidities and endoscopic findings were noted for calculating RS and GBS.

Statistical analysis

Categorical variables were presented as frequencies or percentages and continuous variables as mean±SD. We used Fisher’s exact and Chi-square test to compare and analyze categorical data, and the Student t-test for continuous variables. Values were considered significant if p<0.05 (95% confidence interval). Data collected were managed on a Microsoft Excel sheet, analyzed using the latest version of Microsoft Office 2010 and using GraphPad QuickCalc online (GraphPad Software, San Diego, CA).

## Results

From the total number of 194 patients with UGI bleeding presented, 150 patients (77.31%) belonged to group A, and 44 (22.69%) belonged to group B. Majority of patients in both groups were male (86.11% vs. 75%). The co-morbidities and demographic, clinical, and laboratory profiles of patients of both groups are depicted in Table 1. The percentage of patients with co-morbidities like the previous history of liver disease, history of previous UGI bleed, and corrosive ingestion were higher among group A patients, whereas non-hepatic disease (i.e., cardiac, diabetes, hypertension, respiratory, malignancies diseases) and history of drug intake were higher among elderly patients (group B).

**Table 1:** Characteristics of study patients. n: number of patients, AFI: acute febrile illness, UAP: upper abdominal pain, SpO_2_: saturation of oxygen, bpm: beats per minute, BUN: blood urea nitrogen. Shock is defined as mean arterial blood pressure of <100 mm Hg and tachycardia as pulse rate of >100 beats per minute.

Characteristics	Group A (age <60 years)	Group B (age ≥60 years)	P-value
Number of study patients (n)	150	44	
Age (mean ± SD years)	39.23±10.96	69.34±8.31	P<0.05
Range (years)	(13-58)	(60-85)	
Male/female (n/n)	126/24	33/11	P>0.05
Co-morbidities/diseases in past: n(%)			
Liver disease	103(68.67)	20(45.45)	P<0.05
Previous GI bleed	57(38)	13(29.54)	P>0.05
Diabetics mellitus	06(04)	12(27.27)	P<0.05
Cardiac	01(0.67)	10(22.72)	P<0.05
Neurological	01(0.67)	02(4.54)	P>0.05
Renal disease	05(3.33)	02(4.54)	P>0.05
Hypertension	07(4.67)	13(29.54)	P<0.05
Respiratory	05(3.33)	04(9.09)	P>0.05
H/O corrosive in past	04(2.67)	00(0)	P>0.05
Drug treatment	27(18)	21(47.73)	P<0.05
AFI	21(13.33)	4(9.09)	P>0.05
Acute pancreatitis	01(0.67)	00(0)	P>0.05
Clinical presentation: n(%)			
Hematemesis	96(64)	29(65.9)	P>0.05
Melana	110(73.33)	32(72.72)	P>0.05
Hemetemesis+malena	57(38)	17(38.63)	P>0.05
Hematochezia	10(6.67)	1(2.27)	P>0.05
Syncope+melena	38(25.33)	10(22.73)	P>0.05
Hemetemsis+syncope	27(18)	5(11.36)	P>0.05
UAP	37(24.67)	15(34.09)	P>0.05
≥3 symptoms	35(23.33)	14(31.81)	P>0.05
Addiction: (n/%)			
Smoker	45(30)	7(15.9)	P>0.05
Alcoholic	74(49.33)	10(22.7)	P<0.05
No addiction	70(46.67)	32(72.72)	P<0.05
SPO_2_ (mean±SD)	95.72±5.47	95.16±4.98	P>0.05
SPO_2_ <95% (n)	28	15	P<0.05
Pulse rate (bpm) (mean± SD)	94.8±12.26	90.14±12.43	P<0.05
Pulse rate >100bpm (n/%)	68(45.33)	12(27.27)	P<0.05
Shock (n)	65(43.33)	6(13.63)	P<0.05
Pallor (n)	115(76.66)	25(56.81)	P<0.05
Hemoglobin (g/dL) (mean± SD)	8.04±2.6	8.97±2.77	P<0.05
Platelets (/mm^3^) (mean± SD)	1.76±1.04	1.51±0.45	P>0.05
BUN (mg/dl) (mean± SD)	26.33±18.08	32.76±19.77	P<0.05
Serum creatinine (mg/dl) (mean± SD)	1.62±1.40	1.84±1.54	P>0.05

The commonest presenting symptom of UGI bleed was melena and hematemesis in both groups. Out of 150 patients of group A, 110(73.33%) had isolated melena, 96(64%) had isolated hematemesis while 57(38%) had both hematemesis and melena. In group B patients, isolated melena was seen in 32(72.72%) patients, hematemesis in 29(65.9%), and both hematemesis and melena in 17(38.63%). Isolated hematochezia was more common in group A than in B [10(6.66%) vs. 1(2.27%), p>0.05]. The proportion of patients with tachycardia was 47.22%(n=68) and 27.27%(n=12), that with shock was 43.33%(n=65) and 13.63%(n=6), pallor was 76.67%(n=115) and 56.81%(n=25), and SpO_2_ <95 was 18.66%(n=28) and 34.09%(n=15) in groups A and B, respectively. P-value was <0.05 for all these parameters.

Of the total 150 patients of group A, 98(65.33%) patients had variceal bleeding, and 52(34.67%) patients had non-variceal bleeding. This proportion in group B was 20(45.45%) and 24(54.55%). Overall, the most common isolated cause of bleed was esophageal varices in both groups. Bleeding due to esophageal varices was significantly higher in group A as compared to group B [96(64%) vs. 19(43.18%), p<0.05]. The proportion of patients with gastric varices [17(11.33%) vs. 4(9%)] was similar between groups A and B. Bleeding from peptic ulcer, including gastric and duodenal ulcer was the significant cause of bleeding among group B patients [10(22.72%)] as compared to group A patients [8(5.33%)] (p<0.05). Upper GI malignancy-related UGI bleed was more common in group B, and the result was statistically significant (p-value<0.05; Table 2).

**Table 2:** Causes of acute upper gastrointestinal bleeding - endoscopic findings according to age n (%). EV: esophageal varices, GV: gastric varices, PU: peptic ulcer, GU: gastric ulcer, DU: duodenal ulcer, MVT: Mallory-Weis tear, GAVE: gastric antral vascular ectasia, EMD: esophageal mucosal disease, E ulcer: esophageal ulcer.

Endoscopic profile: (n/%)	Group A (age <60 years)	Group B (age ≥60 years)	P-value
Variceal-	98(65.33)	20(45.45)	P<0.05
EV	96(64)	19(43.18)	P<0.05
GV	17(11.33)	4(9)	P>0.05
Nonvariceal			
PU	8(5.33)	10(22.72)	P<0.05
GU	6(4)	5(11.36)	P>0.05
DU	2(1.33)	5(11.36)	P>0.05
MVT	4(2.67)	0(0)	P>0.05
Malignancy	5(3.33)	6(13.63)	P<0.05
GAVE	23(15.33)	2(4.5)	P>0.05
EMD	19(12.67)	10(22.72)	P>0.05
Polyp	0(0)	1(2.27)	P>0.05
E ulcer	1(0.67)	0(0)	P>0.05
No source located	18(12)	06(13.63)	P>0.05

All patients of both groups were managed using terlipressin, proton pump inhibitors, and/or endotherapy. Surgical therapy was not required in any patients of variceal bleed. The requirement of blood transfusion was in 96(64%) patients of group A and 20(45.45%) in group B (p<0.05).

In our study, mortality was 9.33%(n=14) in group A and 13.63%(n=6) in group B (p-value >0.05). Except for one death in the younger age group, all were due to variceal re-bleed. Whereas in the older group, two deaths were due to variceal bleed, the other two deaths were due to gastric ulcer re-bleed, one due to duodenal ulcer re-bleed, and one due to underlying co-morbidity in a case of a gastric bleed. Fifteen (10%) patients from group A and 6(13.63%) from group B developed re-bleeding in 30 days follow-up. The difference in RS in both groups was statistically not significant (5.02±2.12, 5.98±1.91; p >0.05). RS≥6 was present in 62(41.33%) patients of the younger age group; of them, 13 succumbed to death. Similarly, RS≥6 was seen in 23(52.27%) patients of the older age group and mortality occurred in four patients. Mean GBS was 11.65±4.61 in group A and 10.68±4.65 in group B (p>0.05). GBS ≥10 was found in 107(71.33%) patients of the younger age group, out of which there were 14 deaths. On the other hand, 24(54.54%) patients of the older group had GBS ≥10 and had mortality in five patients (p<0.05; Table 3).

**Table 3:** Management and outcome of upper gastrointestinal bleeding in study patients. RS: Rockall score, GBS: Glasgow Blatchford score.

Management	Group A (age <60 years)	Group B (age ≥60 years)	p-Value
Endoscopic treatment (n/%)	120(80)	36(81.81)	p>0.05
Terlipression (n/%)	95(63.33)	16(36.36)	p<0.05
Blood transfusion (n/%)	96(64)	20(45.45)	p<0.05
Band ligation (n/%)	81(54)	17(38.63)	p>0.05
Endotherapy successful (n/%)	84(56)	17(38.63)	p>0.05
Outcome			
RS (Mean± SD)	5.02±2.12	5.98±1.91	p>0.05
GBS (Mean± SD)	11.65±4.61	10.68±4.65	p>0.05
Re-bleed 30 days (n/%)	15(10)	6(13.63)	p>0.05
Mortality 30 days (n/%)	14(9.33)	6(13.63)	p>0.05
RS>6			
Total number (n)	62 (p<0.05)	29 (p<0.05)	
Mortality (n)	13	4	
GBS>10			
Total number (n)	107	24	
Mortality (n)	14 (p<0.05)	5 (p<0.05)	
Mean duration of hospital stay			
Days (mean± SD)	5.22±2.5	6.16±2.96	p<0.05

## Discussion

Despite many recent advanced techniques in the field of gastroenterology to diagnose the cause of UGI bleed, UGI endoscopy is still the primary modality for the evaluation of UGI bleed. Endoscopy has a sensitivity of 92% and specificity up to 100% [17,18].

In our study, the majority (77.31%) of patients were of <60 years age group and 22.69% of ≥60 years age group. In another study from India, the prevalence of the older (≥60 years) population was 33.15% in patients who presented with UGI bleed. Hematemesis and melena were the most common presenting symptoms. The prevalence of isolated hematochezia in our study was more common in the younger age group patients as compared to the older population (6.66% vs. 2.27%), which is studied by Saurabh et al. (1.9% vs. 29%) [19].

Saurabh et al. also found variceal bleeding as the most common cause and endoscopic finding of UGI bleed in both the groups, but in their study, the prevalence of variceal bleed was low as compare to our study. They also found gastric and duodenal ulcers as a predominant cause of bleeding in the elderly group [19].

We found tachycardia, shock, and blood transfusion (BT) requirement much more common in younger patients. This finding was contrary to Saurabh et al., in which postural hypotension (29.3% vs. 14.9%, p<0.01) and BT requirement (20.2% vs. 10.1%, p<0.01) significantly higher among the elderly group than in the non-elderly group [19]. More prevalence of tachycardia and shock in the younger age group can be explained by more number of younger patients with variceal bleed. Variceal bleed tends to cause profuse bleeding. More number of patients having isolated hematochezia in the younger group can contribute to severe anemia, as hematochezia is the passage of rapid and fresh bleeding per anum. Thirty days re-bleed (group A 10%, group B 13.67%) and mortality rates (group A 9.33%, group B 13.63%) were similar in both groups. Saurabh et al. also had similar re-bleeding rates in both groups but had the mortality rate is significantly higher in elderly patients compared to the non-elderly patients (10.32% vs. 1.94%, p<0.01). They explained this by the presence of multiple co-morbidities and chronic drug intake in these patients [19]. The similar mortality rate in both the groups in our study was probably because of the presence of co-morbidities in both the groups. Younger age group patients had a much higher prevalence of the underlying liver disease, history of previous GI bleed, and history of corrosive injection, whereas the older population had a significantly higher number of patients suffering from non-hepatic disease, i.e., cardiac, diabetes, renal, chronic drug injection, hypertension. The mortality rates among patients aged over 60 years vary from 12% to 35%, while the corresponding rate for patients younger than 60 years of age is <10% [5,20].

Charatcharoenwitthaya et al. studied clinico-endoscopic diagnosis and outcome of UGI bleed between patients aged ≥65 years compared with those aged <65 years and found peptic ulcer bleed as a predominant cause followed by varices and gastropathy. They noticed hemodynamic instability less in the elderly group, contrary to our study. They found a similar clinical course with regard to the utilization of endoscopic therapy, the requirement for transfusion, duration of hospital stay, need for surgery, rebleeding, and mortality between younger and elderly age group patients [21]. Theocharis et al. found peptic ulcers as the main cause of bleeding in patients aged more than 65 years. Co-morbidity, in-hospital complications, and deaths were more common in octogenarians and the severe co-morbidity was the main adverse factor for clinical outcome and mortality [22].

The less prevalence of peptic ulcer disease in our study may be due to lesser use of NSAIDs, and eradication of *Helicobacter pylori* infection. A literature search showed re-bleeding rate varies widely and ranges from 20% to 30% and is affected by multiple factors. This study shows that the trend of UGI bleeding in western Rajasthan is different from the developed countries as UK Audit 2007 has reported only 11% bleeding varices and Snaders et al. have reported only 4.4% [23,24]. The majority of younger age group patients who died had re-bleeding and had underlying liver disease and a history of previous GI bleed. It appears that co-morbid illness predisposes for re-bleed.

## Conclusions

In conclusion, we found that nearly 22% of patients who presented with UGI bleed belong to the age group ≥60 years. Clinical presentation of UGI bleed in both age groups was almost similar with variceal bleeding being the most common cause. Hemodynamic instability was more in the younger age group probably due to more number of younger patients with variceal bleed with more severe anemia, shock, and hematochezia. The presence of multiple co-morbidities in both groups kept 30 days mortality and re-bleed rates similar. Hence, it is a clinical challenge to deal with younger as well as elderly patients for proper management and prevention of underlying diseases, comorbidities, and risk factors to prevent UGI bleed.
